# Plant Foods as Healthy Sources of Dietary Fibre, Microbiota Modulation and Bioactive Compounds: Beyond Definitions—A Review

**DOI:** 10.3390/nu18121957

**Published:** 2026-06-17

**Authors:** Isabel Goñi, Araceli Redondo-Cuenca

**Affiliations:** Department of Nutrition and Food Science, Faculty of Pharmacy, Complutense University of Madrid, Plaza Ramón y Cajal s/n, Ciudad Universitaria, 28040 Madrid, Spain

**Keywords:** DF complex, bioactive compounds, polyphenolic bioavailability, healthy diet, gut microbiota

## Abstract

Dietary fibre (DF) and bioactive compounds (BCs) are essential components of a healthy diet and are abundant in plant-rich dietary patterns. Increasing evidence demonstrates that their combined and synergistic actions significantly influence human health, largely through their effects on the gut microbiota. This review highlights the need for more precise terminology regarding DF and BCs, as inconsistent use of these terms can create confusion among both consumers and researchers. The DF complex encompasses all non-digestible food components that have a positive effect on human health, together with the BCs associated with them, recognising that DF often serves as a carrier for these compounds throughout the digestive tract. Although recommended intakes for BCs have not been established, intake levels observed in populations adhering to healthy dietary patterns may serve as useful reference points. Updated data on the intake and estimated intestinal bioaccessibility of polyphenolic compounds in the contemporary Spanish diet are presented.

## 1. Introduction

The scientific connection between diet and health has been well documented for many decades, with substantial and increasingly robust evidence suggesting that a healthy lifestyle (including adherence to a healthy dietary pattern) is associated with individuals maintaining good health and may be associated with a reduced risk of chronic diseases at all stages of life. The core elements of a healthy dietary pattern are remarkably consistent across the lifespan and are associated with multiple health outcomes. The Dietary Guidelines for Americans 2025–2030 [1] focus their recommendations on consuming a healthy dietary pattern and emphasise the importance of the overall pattern, rather than isolated nutrients, foods, or food groups. A dietary pattern is defined as the usual combination of foods and beverages that constitute an individual’s complete daily intake within a social group [2]. It is therefore all the components of food consumed that act synergistically in the body and are associated with health of the consumer.

All foods in the diet obviously contribute to this synergistic effect, but some food groups such as plant-based foods, and especially fruits and vegetables, are widely recognized as healthy. This suggests that changes in dietary behaviour and lifestyle, such as increasing the consumption of fruits and vegetables and engaging in regular physical activity, may be associated with a reduced incidence of chronic diseases [3].

FAO and WHO [4] jointly held an international expert consultation on sustainable and healthy diets. The consultation agreed on guiding principles for “Sustainable Healthy Diets”, based on foods, nutrient-based dietary guidelines, and environmental, social, cultural, and economic sustainability. The guiding principles of this health-related consultation define the characteristics of a healthy pattern, highlighting that a varied diet with significant amounts of different plant food groups and a limited number of processed foods provides the components (nutrients and non-nutrients) associated with good health and the enjoyment of well-being.

Environmental risk factors include tobacco use, insufficient physical activity, excessive alcohol consumption, and diets high in salt, sugar, and fats, particularly saturated fats. Unhealthy diets and lack of physical activity may be associated with raised blood pressure, elevated blood glucose, increased blood lipids, and obesity, among other harmful effects. These are termed metabolic risk factors and are associated with cardiovascular disease, the leading non-communicable disease in terms of premature deaths.

Conversely, several healthy dietary patterns exist around the world, such as the DASH diet (Dietary Approaches to Stop Hypertension), the Mediterranean diet, the MIND diet (Mediterranean-DASH Intervention for Neurodegenerative Delay), the traditional Asian diet, and the Nordic diet. All of these share common characteristics: a higher content of plant-based foods (fruits, vegetables, whole grains, legumes, seeds, nuts) and a lower intake of foods of animal origin (fatty and processed meats), which are associated with favourable health outcomes [5].

Public health guidelines from various organisations recommend a minimum daily intake of five servings of fruits and vegetables. The Eurodiet core report, the World Cancer Research Fund, and the WHO/FAO recommend at least 400 g/day; Denmark recommends 600 g/day, and the USA 640–800 g/day [6]. Regular fruit and vegetable consumption is associated with favourable health outcomes. However, a significant gap remains between recommended and actual consumption. According to Lee et al. [7], only one in ten adults meets daily fruit and vegetable intake recommendations.

There is increasing evidence to suggest that the health benefits of plant-derived foods may be explained by the synergy or interactions between bioactive compounds (BCs) and nutrients in whole foods. Therefore, consumers should obtain nutrients and BCs from a balanced diet with a wide variety of plant foods for optimal nutrition, health, and well-being, rather than from dietary supplements [8].

Traditionally, macronutrients and micronutrients have been considered the main components of food. However, in plant-derived foods, the presence of other minor components that are not essential for life but possess properties associated with biological effects relevant to health is widely recognised. Plant foods are generally high in water, vitamins, minerals, and contain significant amounts of dietary fibre (DF) and BCs with biological activities associated with antioxidant-related functions.

Therefore, discussing nutritional content alone to define a healthy dietary pattern is insufficient, as scientific evidence suggests that BCs may contribute to associations with reduced risk of chronic diseases [9]. In this regard, and continuing the line of reasoning outlined above, the health benefits of fruits and vegetables are attributed to synergistic interactions consistent with the hypothesis that nutrients and BCs within foods may jointly contribute to these effects.

Despite numerous high-quality publications offering valuable insights on this topic, the authors of this work have perceived the need to standardise definitions and concepts to facilitate the exchange of high-value information and contribute to advancing knowledge in the scientific fields involved. In this sense, it is useful to clarify, among other terms, the concepts of DF and BC, which are highly relevant to the main objective of this work.

In addition, this work aims to further elucidate the role of DF as a carrier for BCs throughout the digestive tract. Furthermore, updated data on the intake and estimated bioavailability of polyphenolic compounds in the contemporary Spanish diet are presented.

## 2. Dietary Bioactive Compounds: Clarifying Definitions and Related Concepts

Foods are products of various origins that contain nutrients, compounds responsible for organoleptic characteristics, and other substances that are associated with positive effects on health independently of their nutritional value. In some cases, foods may also contain substances associated with negative health effects. Bioactivity, therefore, can be understood in either a positive or negative sense. Within the context of this study, BCs are defined exclusively as those demonstrating beneficial effects. Therefore, in our view, a BC is characterised as one that is associated with a predominantly positive effect on human health, extending beyond its traditional nutritional role.

In the scientific literature, terms such as BCs, phytochemicals, phytonutrients, nutraceuticals, and functional foods frequently appear. The indiscriminate use of these terms can confuse both consumers and researchers. Consequently, these concepts need to be standardised or redefined, ideally within an appropriate legal framework. Vettorazzi et al. [10] noted that the boundaries between these terms are unclear and are often used interchangeably or with subtle differences in meaning. Although commonly employed worldwide, there is no international consensus on their definitions.

According to Frank et al. [11], the most basic dictionary definition of a BC is “having or producing an effect on a living organism”. However, this broad definition is insufficient for contemporary safety assessment frameworks and regulatory standards regarding human health. These authors define bioactive food components as constituents of foods or dietary supplements, other than those required to meet basic nutritional needs, which are associated with changes in health status. Another definition describes BCs as extra-nutritional substances that are biologically active and naturally occurring [12]. Câmara et al. [13] describe food bioactive compounds as all compounds, mostly without nutritional value and naturally present in food, that are associated with biological activity in the human body.

Xavier et al. [14] noted that BCs are present in both natural and processed foods and have potential roles that may be associated with health outcomes. Their roles are described as being associated with modulation of inflammation, antioxidant effects, reducing lipid levels, and regulating gene expression, which are crucial for preventing chronic diseases such as type 2 diabetes and cancer.

Kussmann et al. [15] observed that nature offers a virtually unlimited source of compounds associated with potential health-related effects, termed natural bioactives. These components are classified into four groups: macronutrients (carbohydrates, lipids, proteins), micronutrients (vitamins and minerals), phytonutrients/phytochemicals (terpenes, alkaloids, phenolics, organosulfur compounds), and gut microbiome regulators (probiotics, prebiotics, symbiotics, postbiotics). This classification encompasses all natural components of food. These authors adopt a concept of natural bioactives that differs from most literature, which will be discussed in further detail below.

The origin of a BC is also important. They derive from a variety of natural sources, including plants, microorganisms, animals, and marine organisms [16,17], and may interact with one or more components of living tissue, producing a wide range of effects that are consistent with biological activity in living systems [18,19]. Guaadaoui et al. [18] highlighted that these substances can be natural (terrestrial or aquatic; plant, animal, or other sources such as microorganisms) or synthetic, either partially or fully.

Frank et al. [11] argue that a BC is defined by its biological activity and refers to phytochemicals or animal-derived components with demonstrated activity in biological systems, usually animals and/or humans, without specifying whether the activity is beneficial or harmful. Biesalski et al. [20] noted that biological activity is recognised in compounds associated with positive effects, but this classification is narrow, as negative effects, such as toxicity, allergenicity, or mutagenicity, often dose- and bioavailability-dependent, also represent biological activity that may be associated with adverse outcomes.

In plants, BCs are commonly referred to as phytochemicals. Phytochemicals constitute a heterogeneous group of minor food components, including terpenes, alkaloids, phenolics, and organosulfur compounds. Hoang and Kim [21] and Probst et al. [22] provided a simple definition of phytonutrients as BCs commonly present in plant-based foods such as fruits, vegetables, grains, and teas.

Other authors present a broader but less precise concept, describing phytochemicals as chemical compounds produced by plants through primary and secondary metabolism, which are associated with biological activity [23,24,25]. Lalitha [26] categorises phytochemicals into primary and secondary metabolites according to their role in plant metabolism. Primary metabolites (e.g., carbohydrates, amino acids, proteins, lipids, nucleic acids) are essential for plant growth and basic functions. Secondary metabolites, including alkaloids, glucosinolates, cyanogenic glycosides, phenylpropanoids, flavonoids, and terpenes, are associated with plant defence mechanism and adaptative functions against pathogens, UV light, and herbivores.

Delgado et al. [27] note that primary metabolites (proteins, lipids, carbohydrates) are directly involved in intrinsic metabolic processes, such as growth, development, and reproduction. Secondary metabolites (phytochemicals) are associated with selective advantages to the plant, despite not being part of its main metabolic pathways.

In some studies, the term phytonutrient is used interchangeably, which can cause confusion. According to Delgado et al. [27] phytonutrients may be considered as whole-plant extracts containing one or more phytochemicals. Examples include turmeric, garlic, cinnamon, graviola, and oregano. Phytonutrients are plant foods containing multiple natural BCs (phytochemicals) that are associated with specific biological activities. Kussmann et al. [15] and others defined phytonutrients as natural compounds in plant foods such as vegetables, fruits, whole grains, nuts, and legumes, including phenolic compounds, alkaloids, terpenes, and other secondary metabolites [26,28,29,30]. These compounds can act synergistically with other nutrients to exert effects associated with antioxidant, anti-inflammatory, and neuroprotective outcomes. Thus, the terms phytonutrient and phytochemical are largely equivalent. Therefore, a phytonutrient can be defined as a plant-derived BCs (e.g., resveratrol) associated with health-related outcomes [11].

The term “functional food” was introduced in Japanese literature in 1984 to distinguish a tertiary function of foods, distinct from primary (nutrition) and secondary (preference) functions [31]. Functional foods contain biologically and physiologically active compounds are associated with health-related effects beyond basic nutrition [32,33,34,35]. The constituents responsible for functionality are generally termed “bioactive compounds” [23].

Functional foods are also defined by the Functional Food Center/Functional Food Institute [36] as “natural or processed foods that contain biologically active compounds which, in defined, effective, non-toxic amounts, provide a clinically proven and documented health benefit using specific biomarkers, consistent with the hypothesis that they may contribute to promote optimal health, reduce the risk of chronic or viral diseases, and manage their symptoms.” There is no universal regulatory definition, and meanings vary among organisations.

The term “nutraceutical” derived from “nutrition” and “pharmaceutical”. Nutraceuticals are foods or components of foods that are associated with health-related effects, including the prevention and/or treatment of disease [37]. Aronson [31] notes there is no internationally agreed definition of nutraceuticals, functional foods, or similar terms such as “health foods”. These terms are vague and non-discriminatory; evidence suggests they should be replaced with more precise terminology. Vettorazzi et al. [10] similarly highlight that BCs, “nutraceuticals”, and “functional foods” are widely used in industry and by consumers, yet the boundaries between them are unclear.

Lupton et al. [38] point out that, unlike traditional nutrients (vitamins, minerals, proteins, essential fatty acids, amino acids) which have dietary reference intake (DRI) values, there is no such evaluative framework for bioactives. DF is one exception.

In conclusion, BCs in plant foods are often called phytochemicals, while some authors use the term phytonutrients. Epidemiological evidence shows that higher intake of BCs, such as in the Mediterranean diet, is associated with lower prevalence of cardiovascular disease, cancer, diabetes, and neurodegenerative diseases. Consequently, functional foods containing health-protective components are increasingly popular [13].

The spectrum of food BCs exhibits substantial diversity in origin, structure, and bioactivity that is associated with different physiological outcomes [13]. Prominent examples in fruits and vegetables include phenolic compounds (flavonoids, tannins, phenolic acids, stilbenes, lignans), terpenes and terpenoids (carotenoids, phytosterols), glucosinolates, and alkaloids [12,13,15,23,27]. Lalitha [26] also considers saponins, polysaccharides, and DF as plant-derived bioactive components. Cámara et al. [13] support including polysaccharides such as cellulose.

Phenolic compounds in plants occur predominantly in soluble or bound forms. Soluble phenolics are synthesised mainly in the endoplasmic reticulum and accumulate in vegetative (leaves, stems, roots) and reproductive (fruits) organs. Bound phenolics form when soluble compounds translocate to the cell wall and conjugate with macromolecules such as cellulose and proteins via ester and glycosidic bonds [39,40,41].

BCs from animal sources should also be considered, as they are not exclusive to plants. Examples include bioactive peptides (e.g., carnosine in red meat) and polyunsaturated fatty acids (PUFAs, e.g., docosahexaenoic acid—DHA in fish) [13]. Chaudhary & Garg [12] additionally include omega-3 fatty acids, conjugated linolenic acid, L-carnitine, chitin, chitosan, choline, and glucosamine.

Kussmann et al. [15] suggest that bioactive food compounds should also encompass gut microbiome regulators, including probiotics, prebiotics, symbiotics, and postbiotics.

Based on the reviewed literature, we propose the following definition: “BC are chemicals found naturally, mainly in plant-based foods (fruits, vegetables, nuts, oils, whole grains), which are associated with beneficial effects on human health and may contribute to reducing the risk of chronic diseases. Some may be essential nutrients (e.g., certain vitamins), others non-essential (e.g., carotenoids, polyphenols). They modulate biological functions in ways consistent with the hypothesis that they may contribute to optimal health, rather than to prevent a deficiency.”

In light of the diverse and sometimes conflicting definitions in the literature, this study adopts a standardized terminology framework to ensure consistency throughout the manuscript. Phytochemicals and phytonutrients are treated as equivalent terms restricted exclusively to plant-derived BCs (e.g., polyphenols). Functional foods refer to the whole or processed food matrices containing these active components in effective amounts. Lastly, nutraceuticals are strictly defined as isolated or purified BCs delivered in pharmaceutical dosage forms (capsules, tablets, or powders). The operational definitions, structural hierarchies, and boundaries for each term as characterised and applied in this study are explicitly summarised in Table 1.

## 3. Dietary Fibre: Definition and Components: Towards a Physiological Concept

The scientific community generally recognises DF as being associated with human health. Disagreements often arise, however, when attempting to relate the concept of DF to its physiological properties, as the term encompasses a wide variety of chemical structures.

DF is not a single entity; it is far more complex than it initially appears. This complexity may contribute the apparent inconsistencies sometimes described in the literature, as well as the wide range of physicochemical properties and physiological effects associated with fibre intake. The chemical components comprising the fibre complex vary according to the food source, and consequently, the physicochemical and physiological properties and associated health effects are associated with the source of fibre [42]. Dietary fibre encompasses a diverse set of plant-derived compounds, including but not limited to carbohydrates, that resist digestion by human enzymes and are associated with health-promoting effects.

This work does not aim to provide an exhaustive review of the evolution of the fibre concept, but rather to emphasise and deepen understanding that DF does not correspond to a single type of compound. As such, DF cannot be straightforwardly addressed from either an analytical or nutritional perspective, and its interpretation may depend on the complexity of its constituent structures. Furthermore, the complexity of these structures is compounded by the wide variety of physiological effects associated with the consumption of fibre-containing foods.

As a starting point, we refer to the Codex Alimentarius definition, which is particularly important because Codex sets global standards for food. This definition underpins analytical methods, food labelling, nutrient reference values, and health claims [43]. Codex defines DF as: “Carbohydrate polymers with ten or more monomeric units, which are not hydrolysed by endogenous enzymes in the human small intestine and belong to the following categories: (1) Edible carbohydrate polymers naturally occurring in the food as consumed; (2) Carbohydrate polymers obtained from food raw materials by physical, enzymatic, or chemical means, which have been associated with physiological effects considered beneficial to health according to generally accepted scientific evidence; (3) Synthetic carbohydrate polymers which have been associated with physiological effect considered beneficial to health, according to generally accepted scientific evidence provided to competent authorities.

Stephen et al. [44] support this definition, describing DF as a mixture of qualitatively and quantitatively diverse chemical structures, dependent on the fibre source. Physiological properties are associated with the type and quantity of these structures and their physicochemical properties in the intestinal environment, including viscosity, water-holding capacity, and interactions with minerals, fats, and sugars. On this basis, a classification of DF materials according to the characteristics of their main components may be proposed.

**Figure 1:**
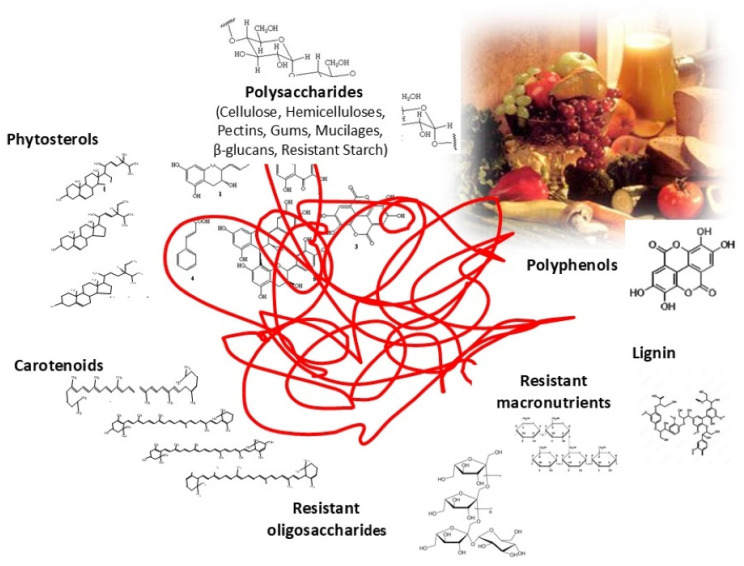
Free representation of the dietary fibre complex, which includes all non-digestible components of plant foods.

Therefore, the concept of DF can be understood as the association of different chemical structures, that are non-digestible in the small intestine. Depending on the plant source, the composition of this mixture varies (Figure 1).

In some fibres, polysaccharides predominate, as in citrus fibre. Others, such as fibres from legumes or red fruits, are often associated with a higher content of polyphenols. In some plant materials, phytosterols predominate, such as in nuts or plant-derived fats, while others, like green leafy vegetables, tend to be associated with higher levels of carotenoids. Each fibre type is structurally distinct, with different physicochemical properties and physiological behaviours, yet all consist of non-digestible components in the small intestine may require specific chemical methods for characterisation.

**Figure 2:**
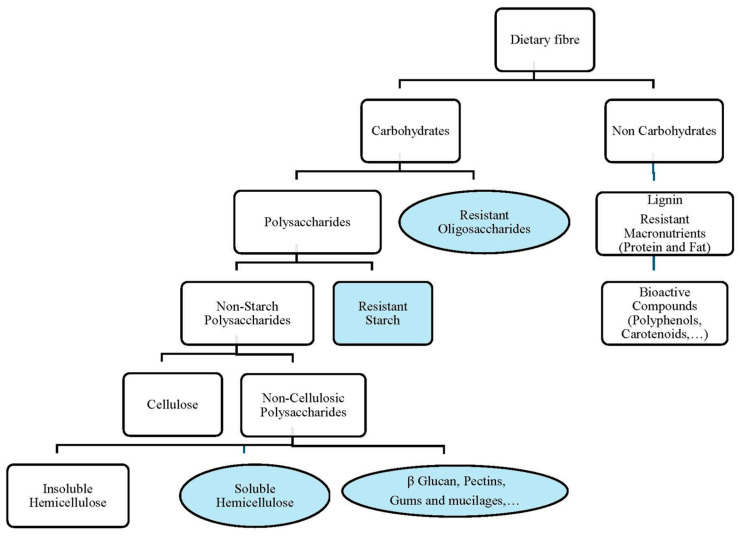
Composition of the Dietary Fibre Complex. Rectangular shapes: Insoluble components; Oval shapes: Soluble components; Shaded: Fermentable components.

As we can see in Figure 2, non-digestible components of foods may include carbohydrates, such as polysaccharides and resistant oligosaccharides. Polysaccharides comprise both non-starch polysaccharides (NSPs) and resistant starch. The NSP fraction includes cellulose, hemicelluloses, β-glucans, pectins, gums, and mucilages. Other non-digestible components not classified as carbohydrates are associated with lignin, undigested macronutrients, and phytochemical compounds such as polyphenols, carotenoids, and phytosterols.

Both carbohydrate and non-carbohydrate fractions contain soluble and insoluble components. Regardless of solubility in the intestinal environment, these non-digestible compounds may be subject to fermentation by the gut microbiota. Generally, soluble compounds are more readily associated with fermentation processes than insoluble ones.

Therefore, the term ‘DF Complex’ is defined herein as all non-digestible components of plant-based foods; this encompasses traditional DF constituents (cellulose, NSPs, resistant starch, lignin, etc.), as well as the associated BCs that contribute to its beneficial physiological effects.

### 3.1. The DF Complex Framework vs. Standard Regulatory Definitions

The central conceptual contribution of this manuscript relies on transitioning from a fragmented view of isolated fibres towards an integrated “DF Complex” framework. It is critical to recognise that standard regulatory definitions worldwide—such as those established by the Codex Alimentarius [45], the European Food Safety Authority (EFSA) [46,47], and the US Food and Drug Administration (FDA) [47], predominantly view dietary fibre through a strict carbohydrate-centric lens (Table 2).

For instance, EFSA explicitly restricts the definition of dietary fibre to non-digestible carbohydrate polymers with three or more monomeric units, completely excluding non-carbohydrate phytochemicals from this classification. While this narrow boundary is highly effective for chemical standardisation and commercial nutritional labelling, it introduces a significant ecological and physiological limitation: it treats plant cell-wall polysaccharides as isolated, pure entities, which does not reflect how they occur in nature or how they behave within the human gastrointestinal tract.

The scientific rationale for our divergence from EFSA and other regulatory definitions, and the justification for including polyphenols and carotenoids within the proposed “DF Complex”, rests upon two foundational pillars:

(1) Structural Co-transport: In intact plant food matrices, carbohydrate polymers do not exist in a chemical vacuum. Hydrophobic and hydrophilic phytochemicals (such as condensed tannins, phenolic acids, and carotenoids) are covalently bound, esterified, or physically entrapped within the polysaccharide cell-wall network. Because human upper gastrointestinal enzymes cannot hydrolyse the carbohydrate skeleton, these bound phytochemicals are effectively protected from stomach acidity and small intestinal absorption. The carbohydrate structures act as a macromolecular vehicle, ensuring the co-transport of these phytochemical fractions intact into the large intestine.
(2) Physiological and Prebiotic Synergy: Once the DF Complex reaches the colon, microbial enzymes ferment the carbohydrate polymer skeleton. This gradual degradation releases the entrapped polyphenols and carotenoids in situ. Rather than acting as isolated compounds, the carbohydrates and the released phytochemicals exert a combined physiological effect. The polysaccharides act as prebiotics to modulate the gut microbiota, while the liberated phytochemicals exert localized antioxidant, anti-inflammatory, and structural signalling effects on the colonic epithelium.

Consequently, evaluating only the isolated carbohydrate polymers while ignoring their naturally bound phytochemical “passengers” misrepresents the true physiological, metabolic, and symbiotic reality of dietary fibre as consumed in whole foods. To provide a clear conceptual advance and delineate our framework from existing paradigms, Table 2 systematically summarises the divergence between international regulatory standards—including Codex, Codex Committee on Nutrition and Foods for Special Dietary Uses (CCNFSDU), EFSA, and US FDA guidelines—and the proposed DF Complex framework.

### 3.2. Methodological Realities and the Indigestible Food Fraction

Saura-Calixto et al. [48] coined the term “indigestible food fraction” for the set of non-digestible components of foods and proposed an analytical method based on physiological conditions in the intestinal tract. In brief, common solid foods in the Spanish diet, including cereals, legumes, vegetables, and fruits, were analysed as consumed. The foods were sequentially incubated with pepsin and α-amylase. Analytical conditions were close to physiological conditions (pH, temperature and incubation times). Following this, they were centrifuged to separate the residue (insoluble fraction), and the supernatants were dialysed against water for 48 h at 25 °C. The soluble and insoluble fractions were quantified separately. Resistant starch, resistant protein, and other associated compounds were included in the quantified indigestible fraction. Consequently, all values quantified as the indigestible fraction were higher compared with those obtained using the AOAC analytical method. Therefore, the estimated intakes based on these data were also higher compared with those typically reported for population groups. The authors concluded by proposing this method as an alternative to the conventional analytical method. This method has been applied to all plant-based foods in the Spanish diet, allowing for the individual characterisation of all components.

DFs are recognised as being associated with gut microbiota composition and function. Fermentable fibres have been shown to be associated with differences in microbiome composition and in the production of beneficial metabolites [49,50,51,52]. Within the DF Complex framework, this microbial modulation is driven not only by the carbohydrate structure but by the synchronised delivery of its structurally associated bioactives.

## 4. The Role of Dietary Fibre in Transporting Phenolic Compounds and Others Bioactive Compounds

As discussed in Section 3, DF does not constitute a single, well-defined chemical group, but rather a combination of chemically heterogeneous substances. The physiological and physicochemical effects of DF are associated with the relative amounts of individual non-digestible components, their interactions with each other, and their integration within the food matrix (Figure 1). In this context, phytochemicals associated with DF may contribute to the physiological properties and health effects attributed to fibre-rich foods [53,54,55].

DF and antioxidants are both recognised as beneficial food constituents and functional ingredients, yet they are often studied separately in chemical and nutritional research. However, a substantial proportion of antioxidants, such as polyphenols and carotenoids [53,56], are naturally associated with DF in plant foods and constitute a major part of the total antioxidants in the diet. Therefore, considering these components together may improve our understanding of the physiological effects associated with fibre-rich foods.

Accordingly, DF and antioxidants could be considered jointly in nutrition and health studies, combining the properties of both in a single material. This approach has led to the concept of antioxidant DF (ADF), defined as a material containing significant amounts of natural antioxidants associated with the fibre matrix. ADF meets the requirements for DF content and intrinsic antioxidant activity derived from the natural constituents of the material, rather than from added antioxidants or compounds released by prior chemical or enzymatic treatments [57]. However, not all fibres exhibit antioxidant activity; only those containing sufficient amounts of antioxidant BCs associated with the fibre matrix confer measurable antioxidant properties. Within the DF Complex framework introduced in Section 3.1, these materials represent examples in which associated bioactive compounds may contribute to the overall physiological properties of the fibre-containing matrix. However, it expands upon this definition by highlighting the significance of minor, non-carbohydrate constituents tightly bound to the matrix, which may be associated with modulation of human health outcomes.

The concept of ADF is useful for distinguishing DF-rich materials with substantial antioxidant capacity from those with negligible activity. Furthermore, the antioxidant capacity of the whole material reflects the cumulative, synergistic activity of polyphenols and other antioxidant constituents. This parameter provides an integrated measure of the antioxidant potential associated with fibre-rich materials and may help explain some of the physiological properties attributed to specific DF sources.

Phenolic compounds, key bioactives in healthy dietary patterns, can be classified according to intestinal bioaccessibility, which depends partly on the composition of the DF complex and its physicochemical characteristics. Two major groups can be distinguished:

(1) Phenolic compounds potentially bioaccessible in the small intestine: These include polyphenols that are solubilised in the stomach and small intestine or released from the food matrix by digestive enzymes. These low-molecular-weight Polyphenols (LPs) could be absorbed, at least partially, through the small intestinal mucosa, followed by metabolism and systemic effects. Only some 5 per cent of the dietary phenols, polyphenols and tannins is absorbed in the duodenum, and of this only some 5 per cent, mainly flavanols, reaches the plasma unchanged, the balance being mammalian conjugates. Over 95 per cent of the intake passes to the colon and is fermented by the gut microbiota [58]; the remainder is considered to be associated with transfer to the colon and is included in the second group.
(2) Phenolic compounds potentially bioaccessible in the large intestine: A large proportion of polyphenols remain unabsorbed along the gastrointestinal tract, accumulating in the large intestine, where they may be extensively metabolised by the gut microbiota [59,60]. These Macromolecular Polyphenols (MPs) include proanthocyanidins and polymeric flavonoids, often associated with other non-digestible food components. The microbiota hydrolyses, reduces, or decarboxylates polyphenols, producing metabolites (e.g., dihydroxyphenyl acids, urolithins, equol) with biological activity [61,62]. The microbiota’s capacity to metabolise non-digestible polyphenols is considered to exceed that of physicochemical or biotechnological treatments, reflecting its complexity and genomic diversity.

In chemical terms, group 1 polyphenols are also called extractable polyphenols, comprising LPs (monomers to decamers) soluble in aqueous organic solvents (methanol, acetone, ethanol, ethyl acetate, etc.). These include flavonoids (flavanols, anthocyanins, flavonols), benzoic and hydroxycinnamic acids, stilbenes, extractable proanthocyanidins, hydrolysable tannins, and others. Non-extractable polyphenols, corresponding to group 2, are MPs and include proanthocyanidins, polymeric flavonoids, and bound to other food components or entrapped within the food matrix, along with minor amounts of carotenoids and other bioactives. These compounds reach the colon, where they interact with the microbiota during colonic fermentation. This process is consistent with the hypothesis that a substantial proportion of the biological activity associated with the consumption of fruits, vegetables, and other plant-derived foods and beverages [63].

To our knowledge, the intestinal microbiota plays a central role in transforming complexes formed by non-digestible food components under physiological conditions, releasing associated compounds that become bioaccessible in the large intestine [64]. Microbial catabolites are often associated with improved absorption profiles compared with their parent compounds due to the colon’s large absorptive area, high luminal concentrations, and specific absorption mechanisms [65].

In summary, only a small fraction of dietary polyphenols is bioaccessible in the small intestine (LP), which are soluble and extractable using organic solvents. The majority (MPs) remain associated with other non-digestible components in the food matrix, often overlooked in bioavailability and metabolism studies, yet they may contribute to biological effects consistent with observed health-related outcomes [55].

**Figure 3:**
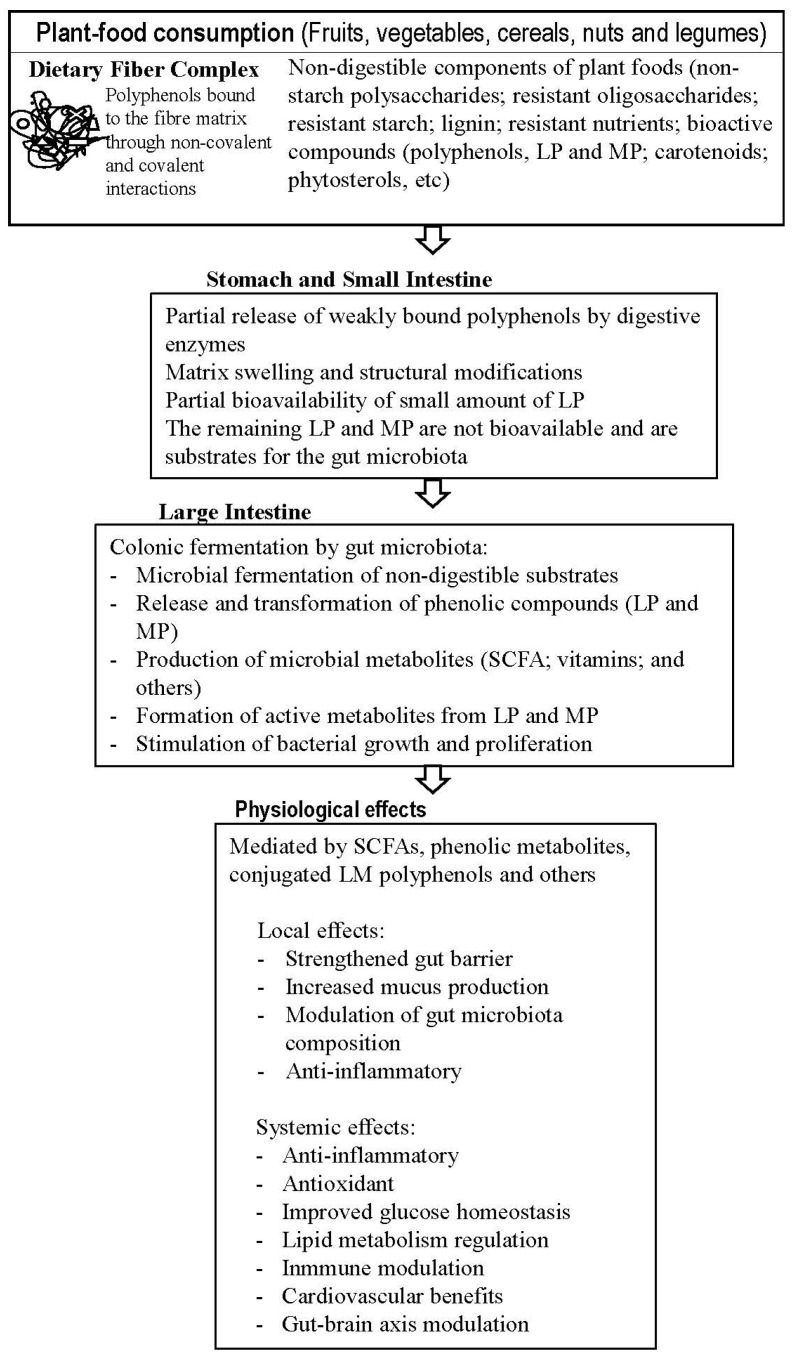
Integrative concept. Dietary fibre complex acts as functional unit determining the fate, intestinal bioaccessibility and biotransformation of its components leading to the production of bioactive metabolites that contribute to local and systemic health benefits.

Figure 3 summarises an integrative mechanistic framework, illustrating the pathways through which the non-digestible components of plant-based foods influence health. The network represents the DF complex.

## 5. The Gut Microbiota: A Metabolic Network Essential for Human Health

### 5.1. Gut Microbiota: A Brief Introduction

The human microbiota comprises all the microorganisms in our body, which can be categorised as commensals, mutualists and pathogens according to their behaviour. Scientific research on the gut microbiota is booming, with experts from different disciplines collaborating to expand our understanding of this vital organ. Our knowledge of the human microbiota has increased considerably since the introduction of 16S rRNA next-generation sequencing (16S rDNA gene). This technological breakthrough has revolutionised our understanding of microbiota composition and its associations with human health [66].

The most densely populated human organ is the colon (10^11^–10^12^ cells g^−1^). It houses more than 70 per cent of all microbes in the human body, including between 500 and 1000 different species. Humans are born essentially sterile and acquire intestinal microorganisms from their mother and the external environment. Microbial colonisation of the gastrointestinal tract associated with various factors, such as mode of delivery, feeding regimen and antibiotic therapy [67].

Interestingly, most of the references consulted indicate that the microbiota genome comprises more than 3.3 million genes, 150 times more than the human genome. Furthermore, each individual microbiome is unique and differs from that of other humans [68]. While the human genome encodes only approximately 25,000 genes, human microbiomes are estimated to encompass between 2 and 20 million genes, representing up to 99.9 per cent of the human body’s genetic capacity [69].

These figures regarding the bacterial population and its proportion relative to the number of human cells have been critically examined using information published by Sender et al. [70]. Recently Dey [71] concluded that the number of gut bacteria is ten times lower than previously predicted based on traditional data, and that the ratio between bacterial cells and human cells is approximately 1:1, constituting a total bacterial mass of 0.2 kg for a typical 70 kg man. This is clearly a very interesting and topical subject for discussion.

The human colonic microbiota can be considered a closely co-evolved microbial partner of the human genome, extending host-encoded functions and potentially enabling the host to obtain energy and other biologically active compounds from food components that would otherwise remain inaccessible and be excreted as waste [68,72].

Microbial communities change dynamically within and between each stage of life, from birth to death [72,73], and respond to the host environment. Most intestinal phylotypes belong to a restricted set of phyla, such as Bacteroidetes, Firmicutes, Proteobacteria, Actinobacteria and Verrucomicrobia [69], but the relative abundance of bacterial phyla and species usually varies in association with external factors, especially diet. Diet is one of the most significant determinants of microbial diversity in the gastrointestinal tract, and dietary components are associated with change in both microbial populations and their distribution [67,69,74].

The entire colonic bacterial population has been described as a “new organ” that possesses marked enzymatic and metabolic activities which are associated with the health of the host [75]. The gut microbiota is equivalent to an internal organ in itself, but it is prone to adaptation and alteration, with potential implications for multiple physiological systems throughout the human body [67]. This includes associations with the liver, brain, pancreas and immune system [73]. These functional effects may be related to the ability of the microbiota to produce various metabolic compounds with bioactive properties, which are transported throughout the body via the circulatory system. There is now no doubt that the composition of the microbiota is strongly associated with human health [73].

The gut microbiota is associated with a wide variety of physiological functions and possesses enzymatic and metabolic activities that may contribute to host nutrition and health.

The physiological effects of the microbiota are thought to arise from three-way interaction between non-digestible food components (substrates), microbiota and epithelial cells (colonocytes), interactions that occur within the intestinal ecosystem continuously throughout life. Bacteria, original substrates, preformed metabolites, metabolic residues and epithelial cells interact in the intestinal environment, and these interactions are associated with numerous effects on the health of both the intestinal ecosystem and the host [73].

Once non-digestible dietary components reach the colon, they become available for the fermentative activities of the colonic microbiota. These compounds have been associated with changes in both species’ composition within the gut microbiota and the metabolite profile in the colon; some metabolites are absorbed, while others remain within the intestinal ecosystem. Interactions, competition and synergistic effects between bacterial enzymes, dietary substrates and metabolites can be expected [76].

Del Chierico et al. [69] proposed a map of the individual gut microbiota based on meta-omics studies of the relationship between consumption of a healthy diet, such as the Mediterranean diet, and the prevalence of disease. Their conclusions are consistent with the comments in previous paragraphs, although most reviewed trials showed considerable variability due to small sample sizes and the wide range of pathologies studied. These are common limitations in many studies exploring the relationship between diet and healthy microbiota balance.

Whether effects are beneficial or detrimental may depends on the balance between bacterial populations. When this balance is altered, dysbiosis occurs and is associated with an increased prevalence of risk factors for disease increase [77]. Dysbiosis refers to an imbalance in the microbial community in the gut and has been associated with diseases such as hepatic steatosis, metabolic syndrome, behavioural abnormalities, metabolic disorders and inflammatory conditions [69,76]. Such imbalance may involve reduced microbial diversity, shifts in the relative abundance of different species, or overgrowth of potentially harmful microorganisms. The appearance of dysbiosis may be influence by the type of substrates used by the microbiota, and it must be remembered that these substrates derive primarily from the diet. Zhang et al. [78] reported that dietary alterations account for 57 per cent of total variation in gut microbiota, whereas genetic background accounts for only 12 per cent.

However, the effects of some of these on the modulation of intestinal ecology and the growth of specific microbial species remain poorly understood.

The range of non-digestible dietary compounds constitutes a heterogeneous group of constituents that corresponds to a broader concept than DF, as it includes not only the classical fibre components but also all food components and/or digestion residues that share a common characteristic: they are not digested in the small intestine.

The DF complex can be fermented by the gut microbiome and thus may contribute to changes in the ecology and metabolism of the bacterial population, as well as interact with epithelial cells. Both metabolites and parent compounds have been associated with alterations in numerous metabolic pathways and may contribute to host health.

Dietary fibres (DFs) have been associated with a wide range of physiological effects that may contribute to gastrointestinal and metabolic health. Their well-established functions include increasing faecal bulk, improving intestinal transit, being associated with modulation of postprandial glycaemic responses, and being associated with lower serum cholesterol concentrations. In addition, fermentable fibres are selectively utilised by the gut microbiota, which is associated with increased production of short-chain fatty acids (SCFAs), including acetate, propionate, and butyrate, which have been associated with intestinal barrier integrity, regulating immune and inflammatory responses, and supporting metabolic homeostasis [50,79]. Recent evidence also suggests that dietary fibre intake may contribute to improvements in glucose homeostasis through microbiota-dependent mechanisms and may be associated with reductions in systemic inflammation [80]. Furthermore, specific fibres such as β-glucans and resistant starch have been associated with enhanced microbial diversity, increased abundance of SCFA-producing bacteria, and improvements in cardiometabolic risk factors [81]. Collectively, these findings are consistent with the hypothesis that DFs are not merely bulking agents, but biologically active compounds with important physiological and microbiome-mediated associations with human health.

Our understanding of the gut microbiome remains limited. There is no agreed definition of what a “healthy” gut microbiome should look like. This hampers our ability to assess the healthiness of gut microbiota profiles based on metagenomic data and makes it difficult to determine what a health-promoting microbiota should look like according to diet and life stage. Current approaches therefore tend to focus on reducing pathological strains while increasing the abundance of health-promoting bacteria such as *Bifidobacterium* and *Lactobacillus* spp. [72].

The increase in certain probiotic bacteria has shown encouraging results in numerous preclinical studies, sparking scientific, industrial and public interest in probiotics and prebiotics (see below) as potential tools for managing and modulating the gut microbiome in consumers and patients. Genomics, bioinformatics and artificial intelligence are helping to identify associations and potential mechanistic links within the intestinal microbial ecosystem and their relationship with host health [82,83,84,85].

### 5.2. Dietary Fibre and Bioactive Compounds: Food for the Gut Microbiota

Despite the enormous relevance of the intestinal microbiota in human health, its study is not the main objective of this work. Therefore, we will focus solely on the study of the non-digestible components of the diet which, due to their indigestible nature, constitute food for the microbiota, or, as Kussmann et al. [15] described them, microbiota regulators.

The set of non-digestible compounds (DF complex) mentioned in the previous sections provides the source of nutrients and fuel for the growth and development of the intestinal microbiota. As indicated above, the composition of these non-digestible compounds can be highly varied, and therefore the metabolites formed and the physiological effects expected will also be diverse. Hence, many studies indicate that DFs are associated with differences in the occurrence of specific bacterial taxa, with this effect varying between individuals. Among the consequences that have been associated with health benefits is microbial diversity, a topic still under debate. Nonetheless, human gut microbial diversity generally responds to diet is associated with health [50,86,87].

It is important to emphasise both the quantity and type of substrates reaching the colon, as these will constitute the “primary food” for the microbiota and may contribute to differences in bacterial proliferation rates. Energy and bioactive metabolites are produced as a result of interactions between substrates and bacteria in the intestinal environment.

The host’s diet is strongly associated with the composition of the gut microbiota [67]. Both microbiota and host maintain a nutritionally symbiotic relationship, and as a result, the gut microbiota has been associated with various host processes, ranging from gastrointestinal immunity to pathophysiological conditions such as inflammatory bowel disease, colorectal cancer, energy metabolism and obesity. Moreover, the gut microbiota may contribute to intestinal mucosal immune responses, barrier reinforcement, and nutrient metabolism [67,86].

The intricate mechanisms underlying the truly symbiotic relationships between the complex array of gut microbes and their mammalian host may not only be associated with the entire bacterial population but, in some cases, specifically with the production of bioactive molecules [67], some of them having been associated with a reduced risk of colon cancer, cardiovascular disease, and immune-mediated disorders. Furthermore, host–microbiota interactions are also associated with the pathogenesis of chronic disorders such as Crohn’s disease, irritable bowel syndrome and ulcerative colitis [88,89,90].

The complexity of fibre matrices—determined by the chemical nature and degree of polymerisation of certain components, and by the presence of oligosaccharides, polysaccharides and other associated compounds such as phytochemicals—is particularly significant in some plant materials. Clearly, there are numerous types of indigestible, fibre-like compounds, and interactions among them naturally occur within foods. Associated BCs—such as polyphenols, carotenoids, terpenes, terpenoids and organosulphur compounds—should be included; these are grouped as phytonutrients in the classification proposed by Kussmann et al. [15].

In conclusion, a variety of substrates (DF complex), depending on the diet consumed, become available for fermentation and may contribute to variations in the metabolic processes occurring within the colon. Some components of complex fibre may exert prebiotic effects, others may have strong antioxidant activity, and yet others may form viscous solutions that are associated with altered glycaemic responses. Therefore, each DF type needs individual evaluation, and specific health benefits must be demonstrated before a commercial product can be offered.

Once non-digestible dietary components reach the colon, they become available to the fermentative activities of the human colonic microbiota. These compounds are associated with changes in both species’ composition within the gut microbiota and the metabolite profile in the colon. Some metabolites will be absorbed, while others remain within the intestinal ecosystem. Interactions, competition and synergistic effects between bacterial enzymes and dietary substrates are expected.

To date, interactions affecting metabolic pathways and numerous metabolites have been widely documented [76,91,92]. Among microbiota-derived metabolites, short-chain fatty acids (SCFAs) are particularly important, as they are predominant metabolites whose effects on the environmental conditions of the intestinal ecosystem, systemic inflammatory processes, and colonic wall permeability have been widely associated with physiological functions [72,79,93]. Dietary tryptophan is also metabolised and converted into indole, indole-3-acetate, indole-3-propionate, tryptamine, and kynurenine, which have been associated with brain connectivity, intestinal permeability, and neuroinflammation [94]. Recent meta-analyses have demonstrated that certain non-digestible substrates, such as prebiotics and phytochemicals, may reduce serum trimethylamine N-oxide trough mechanism that may involve modulation of the gut microbiota [95] and may also be associated with alterations in epigenetic regulation and methionine metabolism [96]. Although PUFAs, particularly omega-3 fatty acids, are not bacterial metabolites [97], a bidirectional interaction exists between the microbiota and PUFAs. Among other effects, omega-3 fatty acids have been associated with increase abundance of SCFA-producing bacteria, reduced markers of intestinal inflammation, and greater microbial diversity [98].

Colonic fermentation is an anaerobic breakdown of substrates by microbial metabolic activity. In addition to processing diet-derived material, the gut microbiota can perform a range of biotransformations on colonic substrates, which may influence their absorption and bioavailability.

The treasure of natural bioactives should be investigated and utilized more efficiently and sustainably for the benefit of human and planetary health. Natural BCs may contribute to human and animal health and may support the development of a healthier, more sustainable food system. Evidence suggests that these approaches may contribute to a more efficient and affordable healthcare system and a more careful use of terrestrial and marine food resources.

Microbiome regulators include health-promoting bacterial strains known as probiotics, as well as edible food components that may influence the composition and activity of the gut flora, referred to as prebiotics. These prebiotic compounds include some components of the non-digestible set—among them phenolic compounds—overlapping with macronutrient and phytonutrient categories. Additionally, optimised combinations of prebiotics and probiotics, known as symbiotics, as well as postbiotic products (including microbially derived short-chain fatty acids, enzymes and vitamins), are also considered part of the microbiome regulator class. Advances in bioanalytical techniques and systems nutrition science provide new opportunities to study these compounds, combining traditional empirical knowledge with modern scientific approaches to elucidate the role of non-digestible food components in human and planetary health [99].

In the past, the concept of prebiotics was limited to non-digestible carbohydrates, but recent evidence suggests that polyphenols also possess prebiotic activity. Indeed, the prebiotic effect may be enhanced when substantial amounts of dietary fibre complex are present. Therefore, regular consumption of a diet rich in plant-based foods is associated with a more favourable gut microbial ecology and may contribute to gastrointestinal health and overall host well-being.

It is increasingly evident that, in order to interpret the role of dietary components in population health, in addition to the necessary epidemiological data, it is essential to establish a new descriptive definition of the set of non-digestible components reaching the colon. Furthermore, analytical methods must be developed to accurately quantify and characterise this set of substrates. Such information would advance our understanding of the underlying mechanisms that may contribute to their observed health benefits.

## 6. Intake, Bioavailability and Prudent Reference Values of Phenolic Compounds

Several systematic reviews and meta-analyses of both observational and interventional studies have reported that diets rich in BCs are associated with a lower risk of numerous chronic diseases. This evidence suggests that further research efforts aimed at establishing a robust methodological process for defining dietary recommendations specific to BCs [100,101].

As with essential nutrients, some non-essential food components cannot be synthesised by the body. However, because they have been associated with health benefits and are considered part of a healthy dietary pattern, they may be regarded as bioactive food components.

In contrast to essential nutrients, non-essential bioactive food components do not typically produce clearly described clinical deficiency symptoms when their intake is inadequate. Moreover, unlike essential nutrients, it may not be possible to carry out experiments that unequivocally demonstrate cause–effect relationships for non-essential food components, whether provided as pure substances, within foods, or from food extracts [100].

Therefore, there is increasing interest in developing dietary recommendations for BCs. Such recommendations require detailed information on the long-term association between habitual intake and health at the population level, which can only be obtained through large-scale observational studies. Nutritional epidemiology relies heavily on accurate intake estimation. However, current methods involve two steps: (1) quantifying food consumption and (2) converting this information into data on nutrients, BCs and kilocalories using food composition tables or databases. Both approaches present significant limitations and fail to estimate the systemic presence of BCs [100,101,102,103].

Both objectives face considerable challenges. Firstly, estimating the consumption of foods that are sources of BCs is prone to methodological errors; secondly, existing databases describing the content and composition of dietary polyphenols are incomplete and insufficient for accurately determining dietary intake. Furthermore, data estimating the intake of these compounds in whole diets are scarce and often controversial for several reasons that will be outlined below. Together, these limitations make estimating BC intake using population-level epidemiological data a difficult—if not nearly impossible—task. Moreover, to better understand the associations between BCs in human health, it is essential to know not only the amount consumed in the diet but also their bioavailability within the body.

In summary, despite the relevance of understanding BC intake for population health, no established reference values exist for prudent intake of these compounds. Therefore, in the following section we will attempt to provide some information related to these aspects, drawing on our modest experience. We will address three main challenges: (1) determining the intake of polyphenolic compounds in the population, as they represent the major dietary BCs; (2) determining their bioavailability in the body; and (3) establishing reference and/or prudent intake values.

### 6.1. Intake of Phenolic Compounds in Populations

The intake of bioactive compounds is usually estimated using a two-step process, both of which are subject to considerable bias, insufficient specificity and precision, and consequently involve errors that are difficult to eliminate.

First of all, estimating polyphenol intake requires secure and reliable data on food consumption within each population. Dietary and supplement data are usually obtained from 24 h dietary recalls and/or food frequency questionnaires [104]. However, a homogeneous protocol for data collection would be necessary, as many uncontrolled factors are associated with the final estimation of food consumption data.

On the other hand, the choice of databases is another critical factor when estimating polyphenol intake. The most commonly used are the United States Department of Agriculture [105] and Phenol-Explorer [106].

These databases have improved considerably in recent years and increasingly provide more precise and detailed information on polyphenol composition. However, their use still presents important limitations. Although they provide information on a wide range of foods, the list does not include all food and polyphenol sources. In addition, the effects of seasonality, storage, and food processing must be taken into account, as these may contribute to variability in the estimates obtained [103].

Experimental variability in food consumption estimates is often assumed. However, food composition data are rarely questioned. Most databases provide information based only on average food composition, not actual composition [101]. Moreover, literature data on the content and composition of dietary polyphenols are partial and insufficient for determining true dietary intake. While the content of certain phenolic compounds in many common foods is reported, comprehensive data on their levels in complete diets are lacking [103].

Another important limitation in the case of polyphenolic compounds is that databases do not include MPs, which may contribute to a general underestimation of intake [107,108]. Data on dietary polyphenols generally correspond to several categories of compounds that we have included in the group referred to as LPs in previous sections of this work. These are quantified in aqueous–organic extracts of foods, while the remaining polyphenols present in extraction residues are usually ignored. This may represent a significant source of error in intake estimation, as these unextracted polyphenols are quantitatively higher than LPs. They are potentially degraded by the enzymatic activity of the gut microbiota and are partially absorbed systemically [48,109]. This is relevant since these compounds have been associated with potential health benefits, possibly through the production of microbiota-derived metabolites [110]. Significant amounts of MPs have been reported in specific plant foods [111,112] and in complete diets [55,113]. Goñi et al. [111] quantified DF as the indigestible fraction following the method described above, as well as the components of each fraction (insoluble and soluble), in fruits, vegetables, and cereals consumed in the Spanish diet. They analysed 16 plant-based foods (almond, onion, lettuce, tomato, cherry, orange, mandarin, cashew, apple, pear, acerola, plum, strawberry, peas, green beans, and dark chocolate) and nine beverages (orange and apple juice, beer, red wine, tea, cider, coffee, coconut milk, and yerba mate). To demonstrate the presence of polyphenols in the indigestible fraction, the concentration of phenolic compounds was measured in the soluble and insoluble fractions isolated from the investigated plant-based foods, including phenol-rich beverages. The results showed that polyphenols are important components of the non-digestible fraction, representing between 1.4 per cent and 50.7 per cent (dry weight) of the insoluble fraction in plant-based foods and between 2.9 per cent and 62.8 per cent of the soluble fraction in common beverages. The total content of phenolic compounds—comprising condensed tannins and hydrolysable polyphenols—ranged from 1.4 per cent to 50.7 per cent (mean: 9.7 per cent in the insoluble fraction) across the analysed samples. The dietary beverages also contained appreciable amounts of the indigestible soluble fraction, ranging from 0.05 to 1.5 g/L, which accounted for an average of 28.65 per cent of the total soluble fraction. These findings are consistent with the hypothesis that polyphenols are significant non-digestible components of common foods.

There is a large body of literature addressing specific types of polyphenols in individual foods; however, studies examining total polyphenols in whole diets are scarce. Therefore, the use of terms such as “total phenolic content” can create confusion when comparing results, as this term may be incomplete if it refers only to LPs. In the present work, total polyphenol content refers to the sum of LPs and MPs.

In a systematic review by Del Bo et al. [101], which included studies conducted in Europe, North America and Asia, the average total polyphenol intake was estimated at 1500 mg/day, 900 mg/day and 800 mg/day in Japan, European countries and North and South America respectively. Several European studies have estimated polyphenol intake in populations following a Mediterranean dietary pattern. These studies share the common feature of using the Folin–Ciocalteu analytical method to determine the polyphenol content in foods, either through direct analysis [114] or by utilising data compiled in the Phenol-Explorer database [115,116,117]. Crucially, this method only quantifies polyphenols present in the extractable fraction (liquid phase), explained previously and referred to as LPs. The mean total polyphenol intake reported in these studies ranged from 700 to 900 mg/day. These values are significantly lower than those reported by authors who also account for the non-extractable fraction (MPs) [55,113].

The intake of polyphenols (LPs plus MPs) was determined several years ago in the complete Spanish diet [55]. The analysis was based on data provided by the food consumption panel published annually by the Spanish Ministry of Agriculture, Fisheries and Food. The food samples used for the analysis of polyphenolic compounds consisted of the daily per capita edible portion of each plant-based food included in the diet. These portions were categorised into five distinct groups: cereals, vegetables, fruits, nuts, and pulses. The samples were sequentially extracted using methanol–water (50:50 *v*/*v*, 50 mL/g of sample, 60 min, room temperature; constant stirring) and acetone–water (70:30 *v*/*v*, 50 mL/g of sample, 60 min, room temperature; constant stirring). Following centrifugation (15 min, 25 °C, 3000× *g*), the supernatants were combined and used to determine the extractable polyphenol content of the original samples via the Folin–Ciocalteu method. This fraction corresponds to the LPs mentioned in this review. In the remaining residues, condensed tannins (proanthocyanidins) and hydrolysable polyphenols were determined separately. These compounds correspond to the MPs mentioned in this review. Polyphenol intake was presented as a range (95 per cent confidence interval) estimated from the analytical data of the foods consumed within each group. For more detailed information, see Saura-Calixto et al. [55]. The mean total polyphenol intake in 2007 was around 3000 mg/person/day [55]. Data updated for 2023, prepared for this document and presented below, indicate that the current estimated intake is 2065 mg/person/day. Although intake has decreased in recent years, both values remain significantly higher than those estimated for other populations mentioned above. Using the same methodology as Saura-Calixto et al., [55] the intake of total polyphenolic compounds (LPs plus MPs, 2196 mg/day) was assessed in an elderly population from a Spanish region [113]. Again, these values are notably higher than population intakes published by other authors [102], which may be attributable, at least in part, to the inclusion of MPs.

### 6.2. Intestinal Bioaccessibility and Bioavailability of Phenolic Compounds

Bioactive compounds are metabolised by digestive enzymes in the small intestine or by the enzymatic activity of the microbiota in the large intestine. The metabolites produced can be absorbed, enter the bloodstream and be distributed systemically throughout the body, or remain within the intestinal environment. Biomarkers of bioactive intake are usually based on the presence of these compounds or their metabolites in blood, urine, or other biospecimens. This process is influenced by the specific absorption, distribution, metabolism, and excretion of each bioactive compound; therefore, their use requires careful validation. This aspect will be discussed in the following section.

Moreover, the intestinal bioaccessibility (defined as the fraction of a compound present in a food or matrix that is released during gastrointestinal digestion and becomes available for absorption in the intestine) and bioavailability (defined as the proportion of an ingested compound that, after absorption, reaches the systemic circulation and is available to exert a biological effect in the body) of each BC varies considerably depending on the type and origin. For example, the most abundant compounds in consumed fruit are not necessarily those that produce the highest concentrations of active metabolites in target tissues [118]. Consequently, to study the role of BCs in human health, it is essential to understand the bioavailability of each compound within the food matrix. Many researchers are investigating these aspects, but they are still not fully understood [118,119,120], and the topic remains an open field of research.

To exert their biological properties, polyphenols must be available to some extent in the target tissue. Food polyphenols must therefore be bioavailable in some form to exert biological effects. The enzymatic release of each compound from the food matrix is one of the key steps in the bioavailability of polyphenols in the gastrointestinal tract. This release may occur in the small intestine and/or the large intestine, or it may not occur at all. Only small polyphenol molecules (LPs) originally present in the food, along with those released from the matrix by digestive enzymes (in the small intestine) or by microbial enzymes (in the colon), may be bioaccessible and therefore potentially bioavailable. Understanding these mechanisms is essential to better interpret potential associations between dietary intake and disease prevalence and to support the estimation of reference intake levels for these compounds.

Several human studies on polyphenol bioavailability have been conducted using isolated compounds rather than whole foods, such as purified quercetin, resveratrol, and catechins administered as single molecules. These approaches have shown that compounds like resveratrol exhibit high absorption but extensive metabolism and low systemic availability [121], while catechins and quercetin glycosides also undergo rapid biotransformation following ingestion [122]. Although these studies are valuable for understanding absorption mechanisms, they may not accurately reflect the bioavailability of polyphenols consumed within complex food matrices [118].

As noted above, the estimated content of MPs in the complete diet is nearly double that of LPs, and the physiological effects of both groups are likely to be associated with their degree of intestinal bioaccessibility. LPs released from the food matrix during digestion—referred to as bioaccessible polyphenols in the small intestine—are soluble in the digesta and susceptible to absorption across the gut barrier. However, most polyphenols are transported through the gut within the non-digestible fraction of food, mainly the insoluble fraction [111,112]. This aspect has been discussed in a previous section. These compounds include condensed tannins and hydrolysable phenolic compounds, as well as a substantial number of small polyphenol molecules (LPs). All of them are resistant to digestion in the small intestine and reach the colon, where they interact with the colonic microbiota. The indigestible soluble fraction may also transport polyphenols to the colon, as these molecules do not cross the intestinal barrier and instead act as fermentation substrates for the microbiota together with other non-digestible dietary constituents [111].

**Figure 4:**
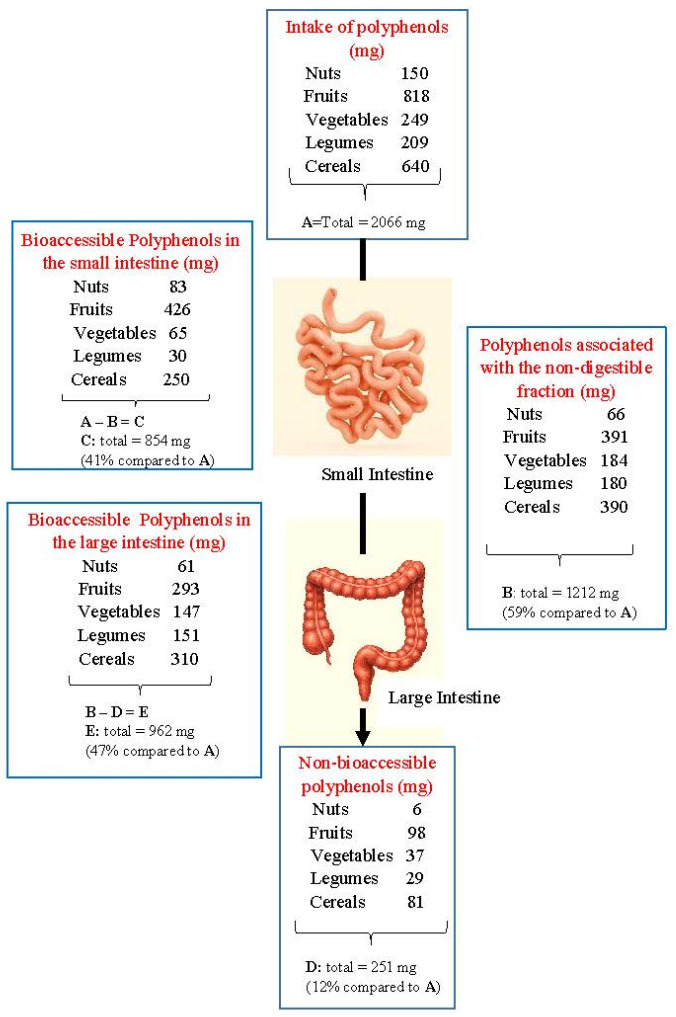
Estimation of the intestinal bioaccessibility of Polyphenols (LP: Low-molecular-weight Polyphenols plus; MP: Macromolecular Polyphenols) consumed from solid plant foods in the Spanish diet, 2023 (mg/person/day).

Saura-Calixto et al. [55] quantified the bioaccessibility of total dietary polyphenolic compounds (LPs and MPs) in the Spanish diet. Using the same methodology, the intestinal bioaccessibility of LPs and MPs in the current Spanish diet (2023) has been estimated and is presented in Figure 4.

An in vitro experimental model was used, and extrapolation to healthy humans. It has many limitations, although it may provide some data of interest. Nonetheless, information on the potential bioaccessibility of different compounds in the small and large intestine is valuable, as the foods analysed were prepared as consumed in a typical Spanish diet, and the experimental conditions simulated digestive and microbial processes occurring throughout the gastrointestinal tract. Moreover, this methodological protocol allows for a more detailed analysis of the metabolites produced in both digestion phases from whole foods [55].

Evidence from this model is therefore consistent with the hypothesis that differences in the intestinal bioaccessibility of LPs and MPs may contribute to variations in their potential physiological relevance, although confirmation in human studies in still required.

It is estimated that approximately 41 per cent of total dietary polyphenols from solid plant foods are bioaccessible in the small intestine. Figure 3 does not include polyphenols from liquid foods, although may be reasonably assumed that polyphenols from beverages are fully bioaccessible in the small intestine, as they pass directly into the intestinal fluids. These would likely represent the main dietary contributors of bioaccessible LPs in the small intestine. Therefore, the values shown in Figure 4 may underestimate the overall amount of bioaccessible polyphenols in the diet.

Other studies have reported very low bioavailability of bioaccessible polyphenols in the small intestine, with values ranging from 5–10 per cent [58]. Thus, evidence suggests that, together with the polyphenols associated with the non-digestible fraction that reach the colon, a substantial proportion of polyphenols potentially bioaccessible in the small intestine may also reach the colon. As shown in Figure 4, 6 per cent of polyphenols ingested from solid foods are potential substrates for colonic fermentation (fraction B).

The current average daily polyphenol intake in the Spanish diet is estimated at around 2066 mg/day (fraction A in Figure 4). Of this amount (LPs and MPs combined), 41 per cent is bioaccessible in the small intestine (fraction C in Figure 4), while 47 per cent is bioaccessible in the colon (fraction E in Figure 4). Only 12 per cent of polyphenols are not bioaccessible in the gastrointestinal tract (fraction D in Figure 4). These data may be useful for the design and interpretation of epidemiological and intervention studies addressing the health effects of polyphenols and plant foods.

Assessing polyphenol bioavailability is of physiological and economic importance, as these ingredients are widely used in the food industry. However, despite the growing body of available data, it remains difficult to draw definitive conclusions about the bioavailability of most dietary polyphenols. Further research is needed on the identification and quantification of their true biological activity, as well as on the development of strategies to improve their bioavailability. Evidence suggests advancing knowledge in this field may contribute to a better understanding of the role of polyphenols in human health and may help inform dietary recommendations for the population. Such findings would be consistent with the hypothesis that differences in polyphenol bioavailability are associated with variations in their potential physiological effects.

### 6.3. Recommended Intakes or Prudent Reference Values for Phenolic Compounds

Recommended nutrient intakes are established on the basis of health problems identified when a dietary deficiency occurs. The adverse physiological or metabolic effects associated with such deficiencies are reversed when the missing nutrient is incorporated into the diet. These dietary standards are thus based on scientific evidence regarding the nutritional needs of virtually all healthy individuals in the population—consistent with the definition of a nutrient. However, this work focuses on dietary BCs, which are not classified as nutrients, although the same procedures may serve as a basis for attempting to establish recommended intakes of BCs.

Whereas RDA/DRI (Recommended Dietary Allowance/Dietary Reference Intakes) committees have defined numerical standards for all known essential nutrients, this has not been the case for BCs. Some countries, such as China and South Korea, have established numerical recommendations for specific bioactives [38]. The South Korean government endorses specific compounds with a generic seal represented by the acronym HFF (Health Functional Foods), which recognises health claims and recommended intakes for 28 essential nutrients and 55 non-nutritive substances. Adequate intake is defined as “the recommended average daily intake level, based on experimentally determined or observed estimates of nutrient intake in a group (or groups) of apparently healthy people”. For instance, the recommended intakes of soy isoflavones and lutein are set at 24–27 mg/day and 10–20 mg/day, respectively.

Meanwhile, the Chinese Nutrition Society has established Recommended Daily Intake (RDI) values for water, fibre, and 18 phytochemicals, assigning them to the SPL (Specific Proposed Level) category based on their efficacy in reducing the risk of non-communicable chronic diseases and improving health status. For example, the SPL values for isoflavones, lutein, and lycopene are 55 mg/day, 10 mg/day, and 18 mg/day, respectively. Crucially, China considers certain BCs to be potentially “life-span essential”—implying that while they are not essential for preventing acute deficiencies, they are vital for achieving a full and healthy life.

Erdman [123] conducted a comprehensive review outlining the development of Recommended Dietary Intakes (RDIs) and Dietary Reference Intakes for BCs. More recently, Yates et al. [124] published a useful framework for developing evidence-based guidelines which have been rigorously reviewed by qualified experts for both efficacy and safety. Their recommendations aim to communicate the intake levels of specific dietary BCs associated with identified health benefits. They proposed a structured, sequential four-step decision-making process and emphasised that “the translation of evidence into recommendations should occur through a structured and transparent process, managed by credible health organisations with expertise and responsibility for developing food and nutrition recommendations”. Yates et al. [124] further highlighted the need to quantify associations between bioactive markers and accepted markers of health or normal function, as well as to determine the safety and toxicity of each bioactive substance, to ultimately translate scientific evidence into quantified intake recommendations.

To date, no standardized process exists for establishing RDI-like recommendations for BCs. This represents a considerable challenge, as demonstrating a relationship between reduced chronic disease risk and intake of a BC is far more complex than identifying disease prevention attributable to deficiency of an essential nutrient [38].

Determining which specific component(s) of a food account for reduced chronic disease risk is equally challenging. Such risk reduction may result from a single BC, from multiple compounds within the same food, or from synergistic interactions between bioactives. Most health-promoting foods contain complex mixtures of bioactive components, rarely dominated by a single compound. Establishing robust evidence consistent with a potential role of an individual bioactive—or a related group—regarding chronic disease reduction is essential for producing meaningful public health recommendations.

In his proposal for establishing RDIs, Erdman [123] underscored the need for rigorous scientific evaluation of both the efficacy and safety of a BC before issuing recommendations for health professionals and consumers. He also stressed that such processes must be conducted by health authorities. At present, evidence suggests that sufficient scientific evidence is available to begin advancing in this field and to establish an appropriate framework for evaluating BCs.

Developing dietary guidelines for the intake of food bioactives within a healthy dietary pattern is an important objective. This process clearly requires at least two types of knowledge: (1) reliable data on food composition and intake to estimate exposure to dietary bioactives, and (2) the ability to assess the intake levels that may be associated with protective effects.

However, given the methodological errors that inevitably occur when estimating BC intake, such estimates become merely indicators of dietary exposure and do not necessarily reflect actual bioactive intake. This makes it difficult to associate estimates with specific health effects and can lead to misleading conclusions in which positive or negative associations between intake and health are incorrectly attributed to BCs themselves rather than to the foods providing them. An alternative approach is the use of nutritional biomarkers to establish reference intakes. Biomarkers rely on the systemic presence of a BC and are therefore not affected by variability in food composition or gaps in food composition databases.

Intake assessments based on nutritional biomarkers represent an advanced alternative, but there are multiple challenges that must be addressed in order to obtain reliable data. Recent reviews have discussed these challenges and highlighted limitations in estimating polyphenol intake and establishing prudent reference values [101,102].

Biomarkers of bioactive intake are typically based on the presence of the compounds or their metabolites in blood, urine, or other biospecimens. Their interpretation, however, depends on compound-specific absorption, distribution, metabolism, and excretion; their development thus requires careful validation. According to Ottaviani et al. [125], nutritional biomarkers are currently the only reliable molecular tools for estimating intake of food bioactives. This field, although already advancing, requires considerable further effort from researchers and institutions. These authors describe the steps involved in identifying and evaluating intake biomarkers for bioactives and highlight common pitfalls and potential solutions.

Although observational studies are essential for identifying potential roles of diet-related compounds, well-controlled and targeted dietary intervention studies—particularly those assessing dose–response relationships—are crucial for identifying a reference or prudent intake (e.g., for health-promoting properties) of food bioactives such as polyphenols, either for the general population or specific vulnerable groups (e.g., the elderly). One advantage of establishing science-based recommendations for selective bioactives is that consumers may be encouraged to increase their intake of plant-based foods if authoritative statements and recommended intake levels are available for bioactives associated with health benefits.

What appears increasingly clear, based on available data, is that dietary patterns rich in BCs are associated with better health outcomes. On this premise, a dietary pattern high in foods rich in BCs may be considered as a potential basis for developing specific recommendations. In this context, Saura-Calixto and Goñi [126] examined the evolution of food consumption in the Spanish diet (Mediterranean diet pattern) between 1964 and 2005 and concluded that this healthy dietary pattern could be defined on the basis of four essential dietary indicators. They provided the ranges of their values considered healthy: (1) monounsaturated to saturated fatty acid ratio (range: 1.6 to 2.0); (2) intake of the DF complex (41 to 62 g/person/day); (3) antioxidant capacity of the whole diet (3500 to 5300 Trolox equivalents/person/day); (4) phytosterol intake (370 to 555 mg/person/day). Indicators 2, 3, and 4 are based on the intake of BCs.

While there is insufficient evidence to recommend precise intake levels, efficacy, or safety for these substances, it is generally agreed that, when consumed as part of a balanced diet, their benefits may contribute positively to health outcomes.

**Figure 5:**
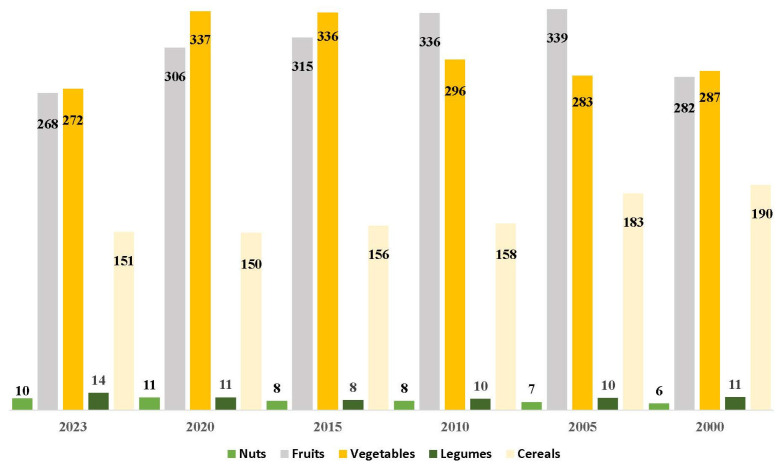
Evolution of household food consumption in the Spanish dies (g/p/d).

Most BCs are found in plant-based foods such as fruits, vegetables, and whole grains, the consumption of which is associated with reduced risk of numerous chronic diseases. The decline in polyphenol intake observed in the American population since 2007 [101] coincides with reduced consumption of fruits, vegetables, and whole grains, a finding consistent with the recommendation to increase intake of these food groups. Similar trends have been observed in other populations, including Spain. Figure 5 shows the evolution of plant-based food consumption (2000–2023) in the Spanish diet. A decrease in the intake of fruits, vegetables, and grains is apparent, similar to trends reported in the American population. Nonetheless, an increase in nut and legume consumption, foods particularly rich in polyphenolic compounds, has also been observed.

Table 3 presents the current estimated intake of polyphenolic compounds (LPs and MPs) in the Spanish population. These values are quite similar to those assessed in the 2005 diet of the same population. Small differences in food consumption (Figure 5) are not reflected in the intake of total polyphenolic compounds (Table 3), as LP intake hardly varies, whereas MP intake declines gradually.

Clearly, much work remains to be done to advance this field. Priorities include: (1) Improving and standardising dietary assessment methods; (2) Standardising analytical procedures for BC analysis; (3) Updating and expanding food composition databases; (4) Establishing validated biomarkers specific to the intake of defined BCs, such as polyphenolic compounds.

## 7. Conclusions

The health benefits of plant-based foods are often reported to be associated with the synergistic effects that arise within a complete diet through interactions between nutrients and non-nutrient components. Among the components potentially contributing to these effects are DF and BCs. These two terms are closely related and sometimes controversial, although both are essential for a healthy diet.

The term DF is generic and non-specific, primarily because it does not refer to a single type of compound but rather to a heterogeneous mixture of chemical components whose composition varies depending on the source. They all share one common property: they are not digested in the small intestine. Based on this idea, the term DF complex has been defined as: “a group of chemical compounds present in plant-based foods that are not digested by endogenous enzymes in the human digestive tract, and which may be associated with beneficial effect on human health”. With regard to BCs, the indiscriminate use of different terminology creates confusion among both consumers and researchers. In this work, the most commonly used terms have been integrated into the following definition: “BC are naturally occurring chemicals, mainly in plant-based foods such as fruits, vegetables, nuts, oils, and whole grains, which have been associated with beneficial effects on human health and contribute to reducing the risk of chronic diseases. Some are essential nutrients (e.g., certain vitamins), while others are non-essential and non-nutrients (e.g., carotenoids and polyphenols). Their role is proposed to modulate biological functions in a manner consistent with the hypothesis that they may contribute to an optimal state of health, rather than to prevent a deficiency”. Most BCs are chemically associated with remaining non-digestible food components, traditionally grouped under the term DF. Fibre compounds therefore act as carriers of BCs, and both are thought to act synergistically in ways consistent with the hypothesis that they may contribute to the maintenance of gut microbiota health, since all non-digestible components present in the large intestine serve as substrates for microbial fermentation. It is important to emphasise the relevance of the quantity and type of substrates that reach the colon, as these constitute the “main food” for the microbiota and may be associated with microbial proliferation rates and overall gut health. Although desirable, there are currently no established reference values for a prudent intake of these compounds, and data on BC intake remain scarce. However, estimating BC intake within healthy dietary patterns could provide indicative figures potentially useful for exploring associations with disease prevalence. For this reason, LPs and MPs intakes and their intestinal bioaccessibility within the current Spanish diet have been assessed.

The current average daily intake of polyphenols in the Spanish diet is estimated at approximately 2066 mg/day, including both of them. This value does not include polyphenols from liquid foods, although most polyphenols from beverages can reasonably be considered to be bioaccessible in the small intestine, as they pass directly into intestinal fluids. These would likely constitute the major dietary contributors of LPs that are bioaccessible in the small intestine. Therefore, the values presented in this work may underestimate the overall intake estimates. The estimated MP content of the complete diet is almost double that of LPs, and the physiological effects of both groups of polyphenols are linked to be associated with their degree of intestinal bioaccessibility. Forty-one per cent of total dietary polyphenols from solid plant foods were bioaccessible in the small intestine, while 47 per cent were bioaccessible in the large intestine. Only 12 per cent were not bioaccessible anywhere in the gastrointestinal tract.

Although these estimations inevitably involve limitations, they represent a valuable contribution to the design and interpretation of studies on the health effects of polyphenols. The analyses were performed using plant-based foods processed according to typical Spanish dietary habits, replicating common patterns of consumption. The experimental conditions for digestive enzymes and the enzymatic activity of colonic microbiota simulated realistic conditions throughout the gastrointestinal tract. Clearly, much remains to be done to advance this field, by both researchers and public health authorities, consistent with the hypothesis that further methodological refinement may improve estimates of bioactive intake and their interpretation.

## Figures and Tables

**Table 1:** Operational definitions and conceptual hierarchy of health-related food components adopted in this study.

Term	Operational Definition	Matrix/Form	Dietary Examples	Main Source/Origin
**Bioactive Compounds (BCs)**	Naturally occurring food constituents that exert a demonstrated, predominantly positive biological effect on human health extending beyond basic nutritional roles.	Universal (present within raw food matrices, processed foods, or isolated fractions).	Polyphenols, bioactive peptides, polyunsaturated fatty acids (PUFAs), prebiotics.	Ubiquitous (plant, animal, marine, or microbial origin).
**Phytochemicals/Phytonutrients**	Bioactive compounds synthesized exclusively by plants through primary or secondary metabolism, often associated with cellular protection.	Plant tissue, whole-plant extracts, or botanical fractions.	Resveratrol, quercetin, lycopene, sulforaphane.	Strictly plant-derived (fruits, vegetables, nuts, whole grains).
**Functional Foods**	Intact or processed food matrices containing effective, non-toxic amounts of bioactive compounds that provide clinically documented health benefits.	Whole or enriched food products.	Oats (containing beta-glucans), fortified milks, fermented dairy.	Plant or animal food matrices.
**Nutraceuticals**	Purified or isolated bioactive compounds extracted from food sources and delivered in specific, non-food medicinal doses.	Pharmaceutical forms (capsules, tablets, pills, powders).	Concentrated green tea extract capsules, fish oil supplements.	Industrial or biotechnological processing of natural sources.

**Table 2:** Comparative analysis of internationally accepted dietary fibre definitions (including CCNFSDU guidance) and the proposed Dietary Fibre Complex framework.

Comparative Criteria	Codex Alimentarius/CCNFSDU [45]	EFSA (European Food Safety Authority) [46]	US FDA (Food and Drug Administration) [47]	Proposed DF Complex Framework
**Structural Scope**	Carbohydrate polymers (≥3 or ≥10 monomeric units, subject to national authorities) naturally occurring, obtained by physical/chemical/enzymatic means, or synthetic. Lignin and associated compounds allowed if intrinsic to the carbohydrate matrix as defined by CCNFSDU guidelines.	Carbohydrate polymers (≥3 or ≥10 monomeric units) including non-digestible oligosaccharides, resistant starch, and chemically associated lignin.	Non-digestible soluble and insoluble carbohydrates (≥3 or ≥10 monomeric units) and lignin natively present in plants, or isolated/synthetic fibres with proven health benefits.	All non-digestible components of plant-based foods; this encompasses traditional DF constituents (cellulose, NSPs, resistant starch, lignin, etc.) as well as the associated BCs that contribute to its beneficial physiological effects.
**Carbohydrate Requirement**	Strict. Grounded in CCNFSDU standards requiring non-digestible carbohydrate structures as the fundamental baseline.	Strict. Exclusively limited to carbohydrate structures and closely bound lignin.	Strict. Limited to non-digestible carbohydrates and isolated fibres meeting clinical endpoints.	Flexible. Carbohydrates form the structural skeleton, but the definition encompasses the whole non-digestible matrix.
**Inclusion of Phytochemicals**	Excluded as independent entities. Under CCNFSDU consensus, secondary metabolites are only acknowledged as minor, unavoidable constituents of the plant cell wall, not as active fibre components.	Excluded. Considered separate bioactive or non-nutritional components.	Excluded. Evaluated under separate regulatory criteria for bioactive ingredients.	Explicitly included. Polyphenols, carotenoids, and other secondary metabolites bound to the matrix are treated as fundamental components.
**Physiological Basis**	Based on non-digestibility in the small intestine and a beneficial physiological effect (e.g., attenuation of blood glucose, laxation) validated by competent authorities via CCNFSDU * scientific criteria.	Based on resistance to digestion and absorption in the small intestine, alongside specific metabolic health outcomes.	Based on physiological attenuation of blood glucose, cholesterol lowering, or improved bowel function.	Based on matrix-driven physiology: structural co-transport, colonic targeted delivery, and symbiotic fermentation (synergy between polysaccharides and bound bioactives).

* CCNFSDU = Codex Committee on Nutrition and Foods for Special Dietary Uses.

**Table 3:** Estimation of the intake of polyphenolic compounds in plant foods in the Spanish diet (mg/person/day).

Food Group	Low Molecular Polyphenols (LPs)	Macromolecular Polymeric Polyphenols (MPPs)	Molecular Hydrolysable Polyphenols (MHPs)
**Nuts**	68.34	15.29	65.82
**Fruits**	178.28	408.60	230.97
**Vegetables**	95.86	-	152.84
**Legumes**	21.30	105.97	82.03
**Cereals**	118.33	-	521.99
**Total**	**482.11**	**529.86**	**1053.65**

## Data Availability

No new data were analyzed in this study. All data presented are mathematical estimates derived from referenced results. Data sharing is not applicable to this article.

## Abbreviations

ADF	Antioxidant Dietary Fibre
AOAC	Association of Official Analytical Chemists
DF	Dietary Fibre
BCs	Bioactive Compounds
CCNFSDU	Codex Committee on Nutrition and Foods for Special Dietary Uses
DRI	Dietary Reference Intakes
FAO	Food and Agriculture Organization
HFF	Health Functional Foods
LPs	Low-molecular-weight Polyphenols
MPs	Macromolecular Polyphenols
MHP	Molecular Hydrolysable Polyphenols
MPP	Macromolecular Polymeric Polyphenols
NSP	Non starch polysaccharides
PUFAs	Polyunsaturated Fatty Acids
RDI	Recommended Daily Intake
RDA	Recommended Dietary Allowance
SCFAs	Short Chain Fatty Acids
SPL	Specific Proposed Level
USA	United States of America
WHO	World Health Organization
